# Entropy generation and thermal analysis of nanofluid flow inside the evacuated tube solar collector

**DOI:** 10.1038/s41598-022-05263-2

**Published:** 2022-01-26

**Authors:** S. Mojtaba Tabarhoseini, M. Sheikholeslami

**Affiliations:** Department of Mechanical Engineering, Babol Noshirvani University of Technology, Babol, Islamic Republic of Iran

**Keywords:** Nanoscience and technology, Nanoscale materials

## Abstract

In the current investigation, the thermal and thermodynamic behavior of a buoyancy-driven evacuated tube solar collector (ETSC) has undergone precise evaluation, and the efficacy of nanoparticle dispersion in the testing fluid was scrutinized. The natural convection process was analyzed in different vertical sections of the absorber tube. The outputs for water and the utilized nanofluid were compared at various cutting planes along the tube during the simulation time. In this problem, CuO nanoparticles with optimum thermal properties were distributed in the base fluid. According to the surveyed results, the temperature distribution analysis illustrates that the mean wall temperature experiences more enhancement when the nanofluid is used. The comparison of the heat transfer coefficient between the two simulated cases show the competency of utilizing CuO-${\mathrm{H}}_{2}\mathrm{O}$ nanofluid and highlight its crucial character in improving the thermal treatment of the operate fluid through the collector pipe. Based on irreversibility assessment, the irreversibility due to fluid friction rises when the nanofluid is applied during the flow time. In contrast, the entropy generation of pure water owing to heat transfer surpasses the case with nanofluid. More specifically, the heat transfer entropy generation experience a reduction of about 6.3% (0.143–0.134 W/K) by utilization of CuO with a volume fraction of 5% after 1 h of flow time, whereas the entropy generation by fluid viscosity enhances up to 23% when the nanofluid is applied in the system. The irreversibility originated from heating and fluid viscosity has significant difference in value, owing to the fluid’s low-velocity range in the natural convection process.

## Introduction

The last decades have witnessed a burgeoning demand for energy consumption produced by fossil fuels, which in turn has led to growing concerns about environmental issues such as air pollution and other serious problems. Take the limited resources of fossil fuels and their high cost as an example. Based on this fact, to alleviate deleterious efficacies in connection with it, researchers have focused their attention and dedicated their study to renewable energy resources. More specifically, solar energy, as an auspicious type of clean energy, is applied in a wide range of applications owing to its accessibility and innocuous usage^1,2^. Solar water and air heaters, solar dryers, desalination systems, etc. are exemplar of solar energy applications in industrial and residential sectors^3^. Solar water heating systems are sweepingly utilized as one of the most prevalent solar energy technologies, which can supply approximately 70% of residential or commercial water heating demand^4^. FPSC and ETSC are including immovable solar collectors by which solar energy can be captured to produce hot water. From a comparative point of view, ETSCs play a more significant role than flat plate collectors because they provide higher thermal efficiency at operating temperatures below 100 ℃^5^. ETSCs are composed of an absorber tube surrounded by two concentric glass tubes that are directly exposed to solar radiation. The vacuum envelope region between the absorber and external tube leads to minimization of the heat loss originating from radiation and convection heat transfer^6^. Moreover, the transmission of the absorbed energy toward the absorber tube occurs by the use of various heat extraction methods^7^. ETSCs are assorted into three types in terms of heat extraction techniques: heat-pipe ETSC, U-pipe ETSC, and water-in-glass ETSC^8^. Water-in-glass or thermosyphon models have been the center of attention of researchers by virtue of low maintenance cost, simplicity, and appropriate thermal performance^9^. Copious numerical and empirical studies were fixated on the treatment of ETSCs. The collector slope, type of working fluids, inlet fluid temperature, and geometrical parameters are the most efficacious factors affecting the collector efficiency^10^. Alfaro-Ayala et al.^11^ carried out optimization for WIGETCs with steady-state and laminar regime using the CFD model and the simulated annealing method. They concluded form optimization analysis that absorber area of the collector’s commercial geometry has been 19.4% lower than case of the optimal geometry with a minimum absorber area of approximately 2.5 ${\mathrm{m}}^{2}$. Other optimum values, such as the number and length of vacuum tubes, were reduced by 40%, while the energy productivity of the whole collector augmented about 26.3%. To increase the performance of the AGETC, Yao et al.^12^ employed twisted tape inside a single-ended evacuated tube for various initial temperatures. The obtained results illustrated that using twist tape is an efficacious method for the system development at the high range of initial temperatures. The intensification of solar flux and its incident angle on the collector performance was performed by Essa and Mostafa^13^ in a numerical transient simulation considering a single-ended tube with a storage manifold. Based on the simulation results, alteration of solar flux intensity leads the flow array to change from the linear profile to the helical form, that originates from the movement of the sun rays. Alfaro-Ayala et al.^14^ performed an investigation changes of the features approaches to evaluate the outlet fluid temperature within the manifold of a WIGETC using CFD simulation. It was achieved that the collector efficiency and outlet fluid temperature were in closer concordance to the experimental data by utilization of the BA method compared to the VPT model. Mazarron et al.^15^ evaluated the fluid flow, design, and installation of a SWH system operating with ETSC at various operational temperatures. Authors proved that the greatest productivity of the proposed unit was obtained for water with T = 50 $^\circ{\rm C}$, and the lowest thermal productivity was for the case with 80 $^\circ{\rm C}$ inlet fluid temperature. Moreover, the profitability of the system decreases, while the operational temperature rises. Apart from all the contributing factors which can develop the performance of the system, using nanofluids is an efficacious technique, leading the solar collectors to have higher thermal efficiency. Owing to the significant capability of nano-fluids for carrying heat up in comparison with other typical fluids, the use of nanofluids has been prevalent in an extensive application, particularly in ETSCs. The thermal functionality of the ETSC has been analyzed in multifarious volume concentration (0.015%, 0.025% and 0.035%) of ${\mathrm{CeO}}_{2}$/water nanofluid by authors of Ref.^16^ who found that, using ${\mathrm{CeO}}_{2}$/water nanofluid with 0.035% volume fraction has shown the greatest influence on the collector efficiency enhancement up to 34%. Ozsoy and Corumlu^17^ analyzed the productivity of a HPETC using silver-water nanofluid. They reported that the proposed system witnessed the efficiency increment about 20–40%, while using nanofluid in place of pure water. Gan et al.^18^ found that employing ${\mathrm{TiO}}_{2}$-water nanofluid under optimum circumstances can lead the productivity of the ETSC to augment around 16.5% in comparison to the case with pure water. Dehaj and Mohiabadi^19^ conducted an experimental study to evaluate the efficiency of HPETC by using MgO nanofluid at different volume fractions. They found that MgO/water nanofluid can enhance the productivity of the collector more than case with water. According to the observations of Yan et al.^20^ in their empirical experiment, ${\mathrm{SiO}}_{2}$/water nanofluid at 5% mass fraction has shown its merits in improving the collector performance compared to plain water. Sharafeldin and Grof^21^ demonstrated the significance and the efficacy of Cu/water nanofluid in ETSC, by examining the nanoparticles at various volume concentrations. The results have divulged that a 50% increment took place in T_out_ while employing the nanofluid at 0.03%, and 0.8 L/min of volume concentration and volume flow rate, respectively.

Regarding the 2nd law, the thermal efficiency of unit will experience a reduction over time, for the reason that the heat energy conversion process is irreversible. The assessment of entropy generation in engineering applications was proposed by Bejan^22^ as an efficacious way to justify the performance improvement in thermal systems, where there are limitations for energy analysis^23^. Employing nanofluids leads the entropy generation of the system to reduce^24^. There are few kinds of literature about ETSCs, which put their main focus on entropy generation and irreversibility. Leong et al.^25^ examined a comparison in terms of entropy generation has been drawn between ${\mathrm{TiO}}_{2}$ and ${\mathrm{Al}}_{2}{\mathrm{O}}_{3}$ nanofluid inside a circular tube. Their observation has shown that the total entropy generation was higher for ${\mathrm{Al}}_{2}{\mathrm{O}}_{3}$ nanofluid than that of ${\mathrm{TiO}}_{2}$ nanofluid. Ramirez-Minguela et al.^26^ appraised the rate of entropy generation for FPC and ETSC using CFD simulation. The obtained results revealed that ETSC has higher thermal entropy generation than FPC by virtue of more heat transmission, which takes place in a vacuum tube collector. In contrast, FPC has shown higher fluid friction entropy generation.

According to the lack of numerical researches concentrating on the appraisal of irreversibility in ETSCs, this study is fixated on analyzing the irreversibly inside an open thermosyphon solar collector aim to extract the efficacy of entropy generation on the productivity of suggested unit. Furthermore, a comparative evaluation between the two cases by utilization of pure water and nanofluid are presented over 1 h of the simulation process. CuO-${\mathrm{H}}_{2}\mathrm{O}$ as one of the most efficacious nanofluids in thermal performance improvement of the solar thermal systems has been selected with optimum properties and volume concentration. In the thermodynamic assessment by consideration of the 2nd law, irreversibility as a result of fluid viscosity and heat transfer are examined and compared for both simulated cases. Moreover, the components of S_gen_ are illustrated throughout the model. For thermal evaluation of the proposed system, temperature and velocity distributions and contours inside the three cutting sections of collector along with the heat transfer coefficient were analyzed for water and nanofluid, by which the merits of using the CuO nanofluid are demonstrated.

## System description and mathematical modeling

**Figure 1:**
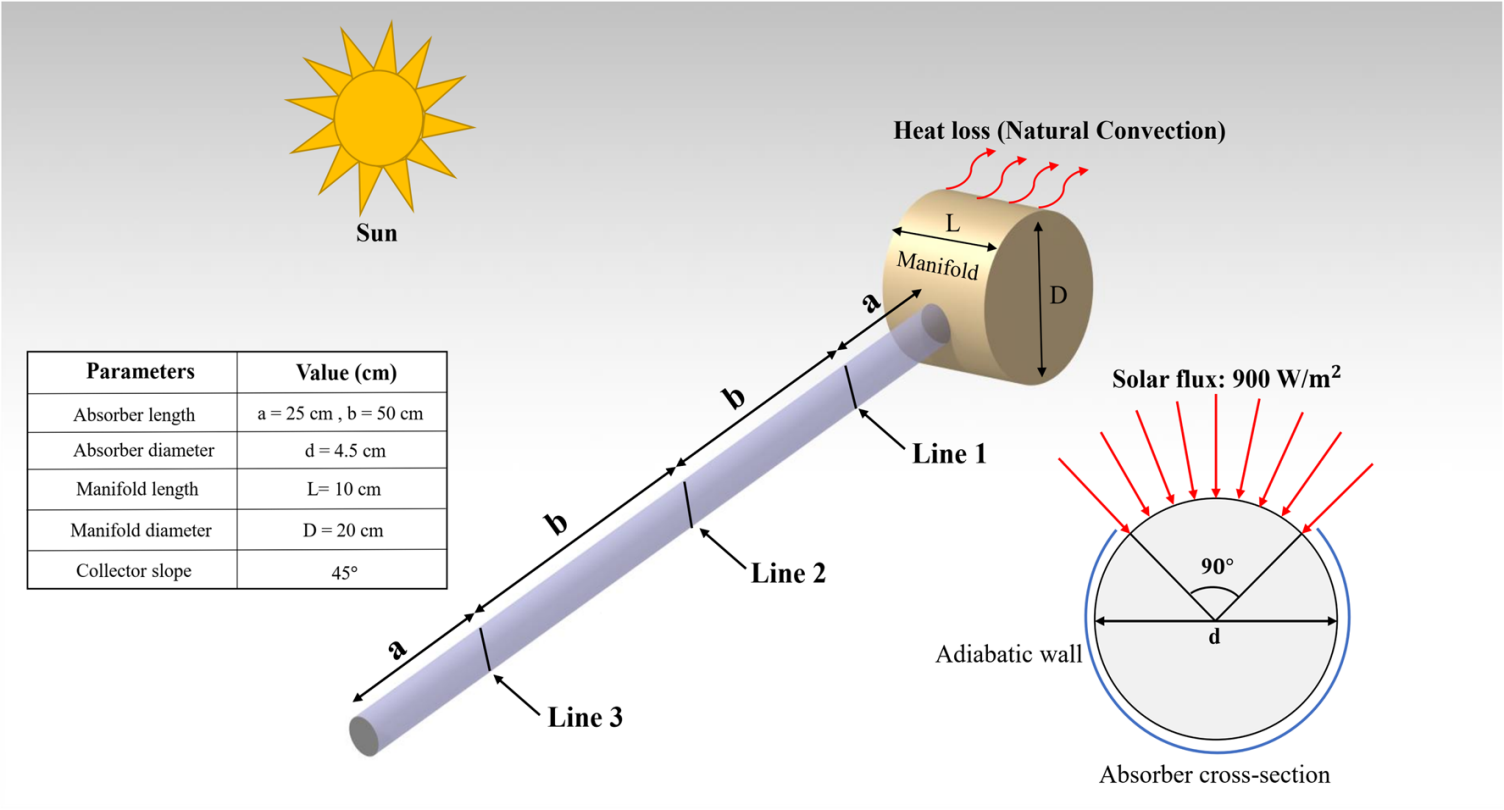
The geometry of the scrutinized solar unit.

The modeling technique has been employed for a close-ended thermosyphon ETSC along with the manifold investigating its thermal behavior and entropy generation. Figure 1 provides an overview of the geometry of the simulated model. As exhibited in this graph, the absorber tube was divided into 3 vertical sections that aim to analyze the flow array within the absorber, from the entrance section toward the close-end. The absorber tube has been considered with a slope of 45 $^\circ$ through the z-direction. The fluid flow simulation process was analyzed using by utilization of the FVM method in ANSYS FLUENT. The terms of velocity and pressure in the governing equations were coupled using the SIMPLE algorithm. What’s more, the Boussinesq approximation method is used for density difference consideration as a result of the slope of the absorber tube. The main assumptions for the fluid flow structure are incompressible, laminar, and continuous. To diminish the computational time and regarding the solution process, which is time-dependent (transient), the modeling time was considered for 1 h.

### Mesh structure, initial, and boundary conditions

**Figure 2:**
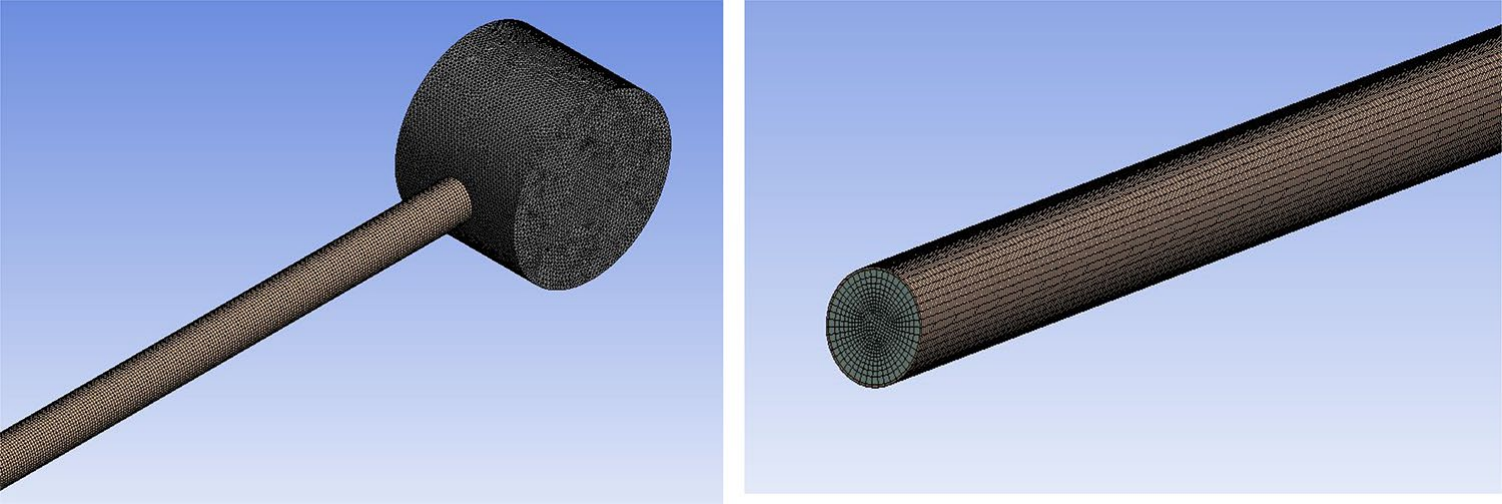
The style of the grid structure.

The mesh structure is effective elements in CFD simulations, on which the correctness of outcome is based. In this investigation, the average tank temperature in different cases with various numbers of nodes varying from 258,743 to approximately 500,000 was compared to reach the optimum number of nodes to reduce computational cost and time. When the number of nodes surpasses 380,000, the changes in the results are trivial and negligible. Hence, 380,675 nodes were specified as the extra fine mesh for modeling. In addition, the value of skewness was 0.17, which shows the high quality of the mesh structure. The shape of the grid applied in current article was illustrated in Fig. 2.

The components of the described model are comprised of the manifold and the absorber tube. There is a heat exchange between reservoir tank and ambient with *h* = 8 W/${\mathrm{m}}^{2}$ K. The tank is at direct exposure to the solar heat flux, which has been assumed as a constant value of 900 W/${\mathrm{m}}^{2}$ during the simulation process. As shown in Fig. 1, part the upper wall of the absorber is specified as the surface on which the solar flux is defined, while other surfaces of the absorber are considered as adiabatic walls. Taking initial conditions into account, the initial velocity and the initial temperature of the fluid were presumed as zero and 30 $^\circ{\rm C}$, respectively. To decrease the computational cost and complexity of the solution, the outer glass tube along with the vacuum envelope between the absorber and the outer tube has been neglected. In order to reach the results with more accuracy compared to the real case, the effects of eliminated parts was involved in boundary conditions^27,28^.

### Validation and time step independence

**Figure 3:**
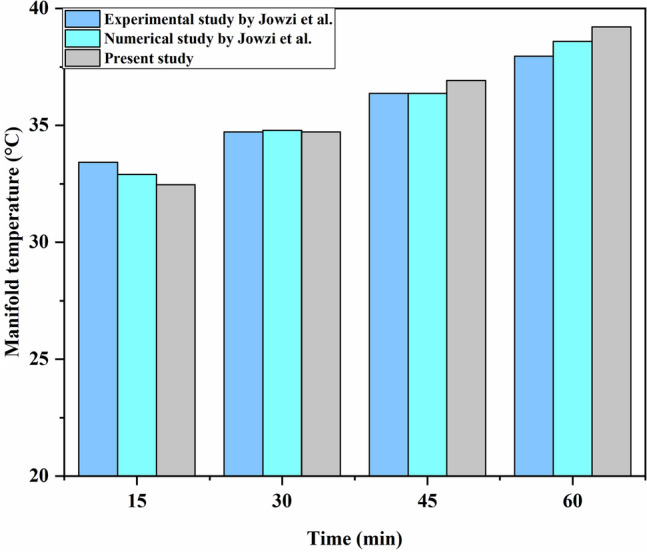
Correctness of the present study based on the numerical and empirical data.

Proving the accuracy of the results is an important part of a numerical simulation. Because of this, the proposed model of Jowzi et al.^7^ has been considered to verify the simulation in the present model. As it is depicted in Fig. 3, the obtained values for the tank’s mean temperature over 1 h of simulation are in good concordance with the numerical and empirical results, with an error of less than 3% in both studies, which shows the authenticity of the current results.

**Figure 4:**
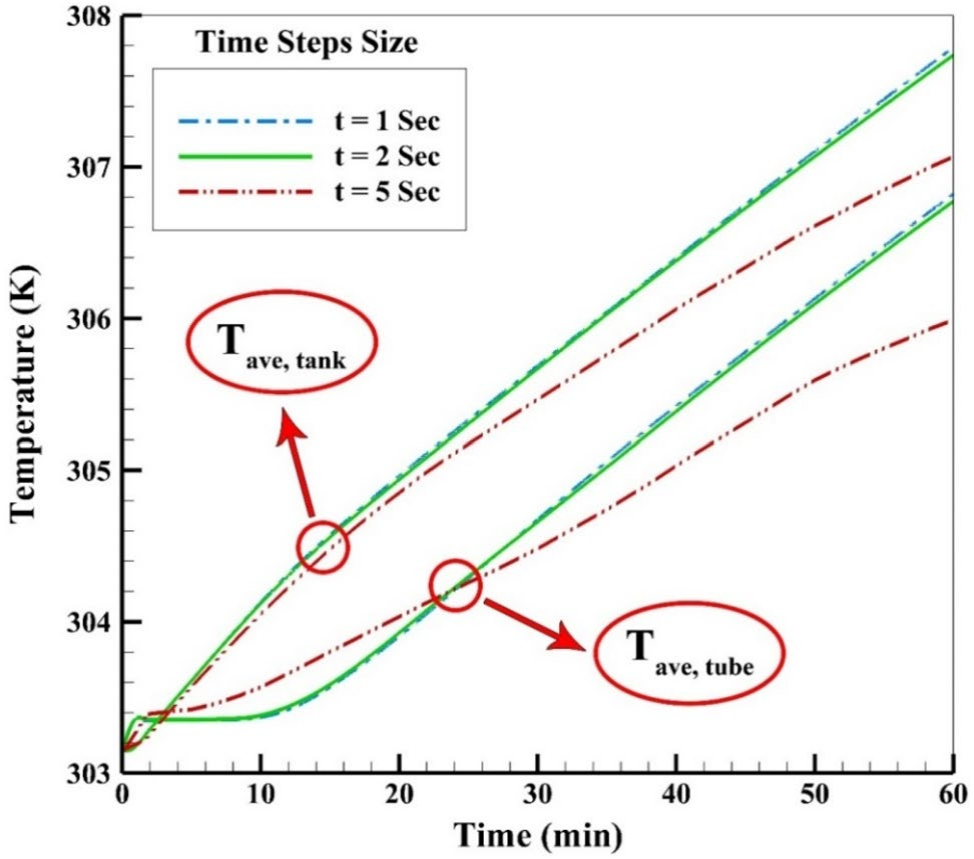
Time step size selection according to the average temperatures.

Choosing an appropriate size for the time step can be helpful to reduce the computational time and simultaneously give accurate results. Figure 4 illustrates the time step analysis considering the mean temperature of the absorber and tank during 1 h of the simulation process. As is obvious, at a 5 s time step size, the T_tube_ and T_tank_ witnesses a significant difference from the outcomes reported at two other time steps after 25 and 15 min of flow time, respectively. Thus, a time step size of 2 s is acceptable for use in the numerical simulation process.

### Mathematical modeling

Aiming to consider lifting efficacy in the modeling, the Boussinesq approximation model has been taken into account. In this approach, density is considered as the variable parameter, while all other thermophysical and transport characteristics are assumed constant. It is stated in terms of temperature (T) and thermal expansion coefficient ($\beta$), which was defined as follows^29^:

1 $$ \rho_{\infty } - \rho = \rho \beta (T - T_{\infty } ) $$

where $\beta$ is the denotation of the volume expansion coefficient with a fixed quantity of 0.000344 ${\text{K}}^{ - 1}$, as shown in Table 1. Considering the buoyancy effect in the conservation equations leads to the equations given below. The continuity equation for the laminar flow regime is defined as follows^7^:

2 $$ \rho \left( {\frac{{\partial u_{x} }}{\partial x} + \frac{{\partial u_{y} }}{\partial y} + \frac{{\partial u_{z} }}{\partial z}} \right) = 0 $$

**Table 1:** CuO nanoparticle and water’s thermophysical specifications used in the present study^34^.

	Cp (J/kg k)	k (W/m k)	$\beta$(1/K)	ρ (kg/${m}^{3}$)	µ (kg/ms)
CuO	531.8	76.50	0.0000180	6320	–
H_2_O	4076.4	0.60475	0.0034112	997.78	0.0009772

The momentum equations are categorized into x, y, and z components^7^:

3 $$ u_{x} \frac{{\partial u_{x} }}{\partial x} + u_{y} \frac{{\partial u_{x} }}{\partial y} + u_{z} \frac{{\partial u_{x} }}{\partial z} = - \frac{1}{\rho }\frac{\partial p}{{\partial x}} + \frac{\mu }{\rho }\left(\frac{{\partial^{2} u_{x} }}{{\partial x^{2} }} + \frac{{\partial^{2} u_{x} }}{{\partial y^{2} }} + \frac{{\partial^{2} u_{x} }}{{\partial z^{2} }}\right) $$

4 $$ g_{y} \beta (T - T_{\infty } ) + \frac{\mu }{\rho }\left(\frac{{\partial^{2} u_{y} }}{{\partial x^{2} }} + \frac{{\partial^{2} u_{y} }}{{\partial y^{2} }} + \frac{{\partial^{2} u_{y} }}{{\partial z^{2} }}\right) = \frac{{\partial u_{z} }}{\partial z}u_{z} + \frac{{\partial u_{y} }}{\partial y}u_{y} + \frac{{\partial u_{y} }}{\partial x}u_{x} $$

5 $$ - \frac{1}{\rho }\frac{\partial p}{{\partial z}} + \frac{\mu }{\rho }\left(\frac{{\partial^{2} u_{z} }}{{\partial x^{2} }} + \frac{{\partial^{2} u_{z} }}{{\partial y^{2} }} + \frac{{\partial^{2} u_{z} }}{{\partial z^{2} }}\right) = \frac{{\partial u_{z} }}{\partial z}u_{z} + \frac{{\partial u_{z} }}{\partial y}u_{y} + \frac{{\partial u_{z} }}{\partial x}u_{x} $$

Energy equation:

6 $$ k\left( {\frac{{\partial^{2} T}}{{\partial z^{2} }} + \frac{{\partial^{2} T}}{{\partial y^{2} }} + \frac{{\partial^{2} T}}{{\partial x^{2} }}} \right) = \rho c\left( {\frac{\partial T}{{\partial z}}u_{z} + \frac{\partial T}{{\partial y}}u_{y} + u_{x} \frac{\partial T}{{\partial x}}} \right) $$

In our study, the suspension of nanoparticles inside pure water was assumed to be the single-phase approach. In this approach, it is assumed that nanoparticles are dispersed into the conventional fluid homogenously. The formulations related to the nanofluid modeling that are applied in this problem are described by the following equations^30^:

Density:

7 $$ (1 - \varphi )\rho_{f} + \rho_{s} \varphi - (\varphi - 1)\rho_{f} = \rho_{nf} $$

Viscosity and heat capacity:

8 $$ (1 - \varphi )^{ - 2.5} \mu_{f} = \mu_{nf} $$

9 $$ \varphi (c_{p} \rho )_{s} - (\rho c_{p} )_{f} (\varphi - 1) = (c_{p} \rho )_{nf} $$

Thermal conductivity:

10 $$ \frac{{ - 2(k_{f} - k_{s} )\varphi k_{f} + 2k_{f} k_{f} + k_{s} k_{f} }}{{k_{s} + \varphi (k_{s} - k_{f} ) + 2k_{f} }} = k_{nf} $$

Thermal expansion coefficient:

11 $$ \beta_{nf} = \left[ {\frac{1}{{1 + \frac{{(1 - \varphi )\rho_{f} }}{{\varphi \rho_{s} }}}}\frac{{\beta_{s} }}{{\beta_{f} }} + \frac{1}{{1 + \frac{\varphi }{1 - \varphi }\frac{{\rho_{s} }}{{\rho_{f} }}}}} \right]\beta_{f} $$

The thermophysical characteristics of CuO nanoparticles were calculated using the above equations, as shown in Table 1. The coefficient of "h" can be achieved as^31^:

12 $$ h = \frac{{q^{\prime\prime}}}{{(T_{w} - T_{b} )}} $$

Turning to the focus of this study, entropy generation analysis has been put forward to assess the treatment of the proposed system. Local entropy generation rate would be obtained from the *V* and *T* fields gained from the CFD analysis by utilization of the thermodynamics second law. The summation of irreversibility owing to fluid viscosity and heating provides the total value of irreversibility as follows^32^:

13 $$ \begin{aligned} S_{gen,l} = & S_{gen,lf} + S_{gen,lth} \\ = & T^{ - 1} \mu_{nf} \left\{ {\left[ {\left( {\frac{{\partial u_{y} }}{\partial y}} \right)^{2} + \left( {\frac{{\partial u_{x} }}{\partial x}} \right)^{2} + \left( {\frac{{\partial u_{z} }}{\partial z}} \right)^{2} } \right]\left( 2 \right) + \left( {\frac{{\partial u_{x} }}{\partial y} + \frac{{\partial u_{y} }}{\partial x}} \right)^{2} + \left( {\frac{{\partial u_{x} }}{\partial z} + \frac{{\partial u_{z} }}{\partial x}} \right)^{2} + \left( {\frac{{\partial u_{y} }}{\partial z} + \frac{{\partial u_{z} }}{\partial y}} \right)^{2} } \right\} \\ & + k_{nf} \left[ {\left( {\frac{\partial T}{{\partial z}}} \right)^{2} + \left( {\frac{\partial T}{{\partial y}}} \right)^{2} + \left( {\frac{\partial T}{{\partial x}}} \right)^{2} } \right]T^{ - 2} \\ \end{aligned} $$

Regarding the equations germane to the nanofluid flow, the entropy generation correlations can be expressed as^33^:

14 $$ S_{gen,f} = \int {S_{gen,lf} } dV,S_{gen,th} = \int {S_{gen,lth} } dV $$

## Results and discussion

### Thermal examination

Current part delineates the impacts of nanomaterial migration and entropy generation inside a single-ended ETSC. The collector tube has been divided into three sections by vertical lines (lines 1, 2, and 3), each of which was surrounded by cutting planes. To reduce the computational cost, the numerical simulation results have been considered for 1 h of flow time, which can be extended to a one-day duration. In the first step, the velocity distributions of fluid flow with plain water and nanofluid are compared in the entire tube and each cutting section after 30 and 60 min, respectively. In the second step, the same comparative analysis has been conducted for the temperature range within the unit for both fluid flows. Finally, entropy generation assessment is carried out for both cases.

**Figure 5:**
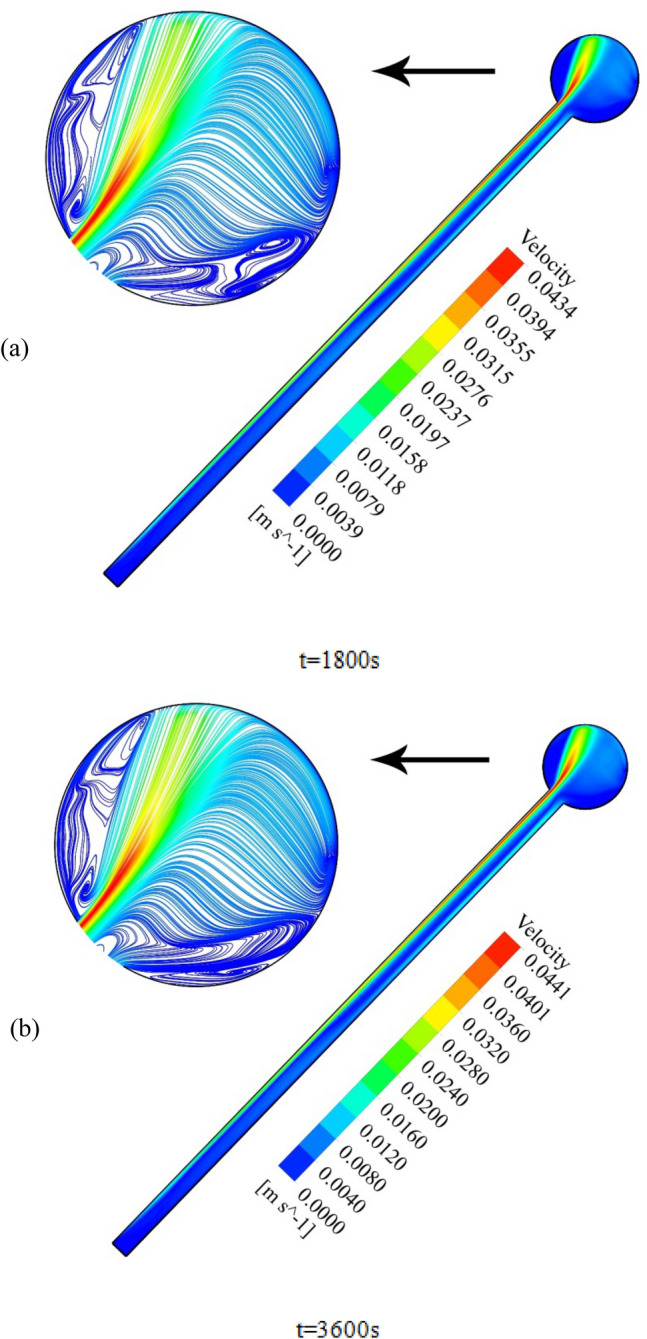
Reporting of velocity for water.

**Figure 6:**
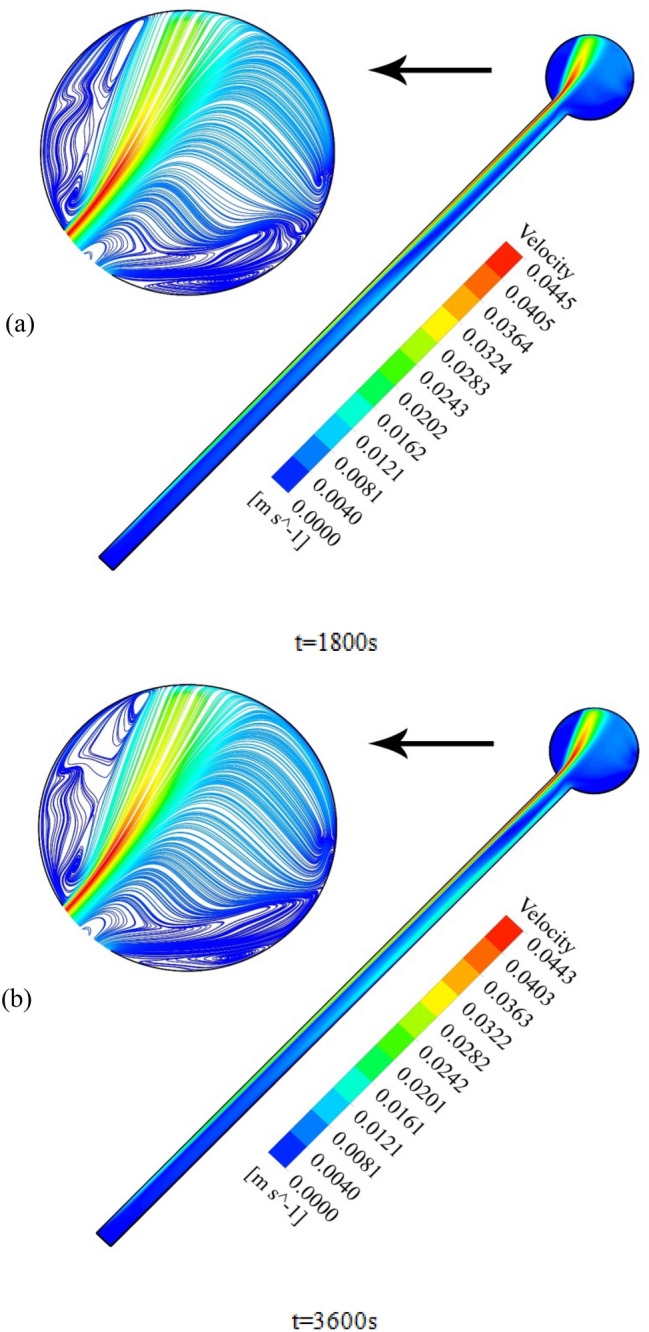
Reporting of velocity for nanofluid.

**Figure 7:**
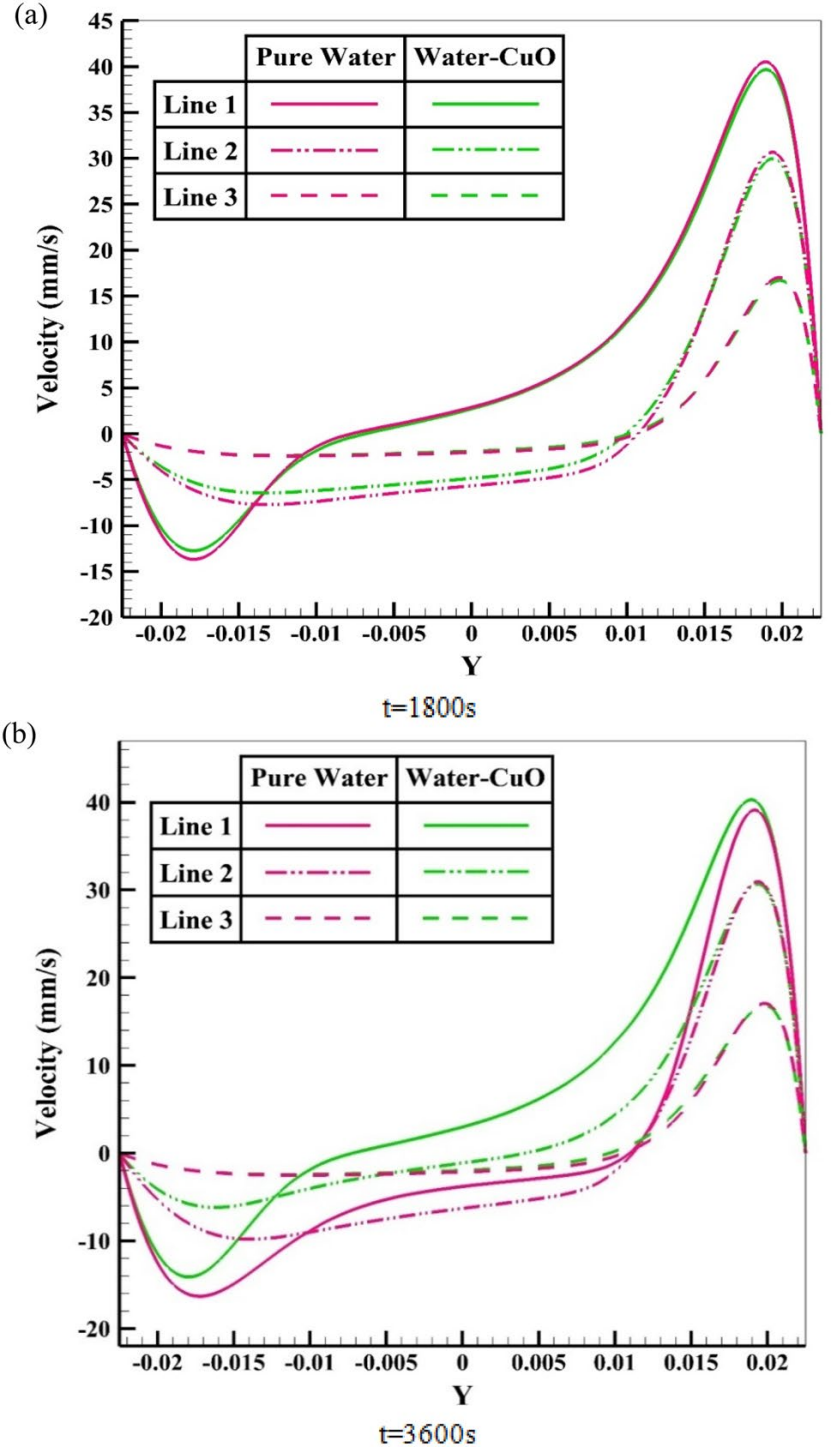
Distribution of velocity at various sections.

**Figure 8:**
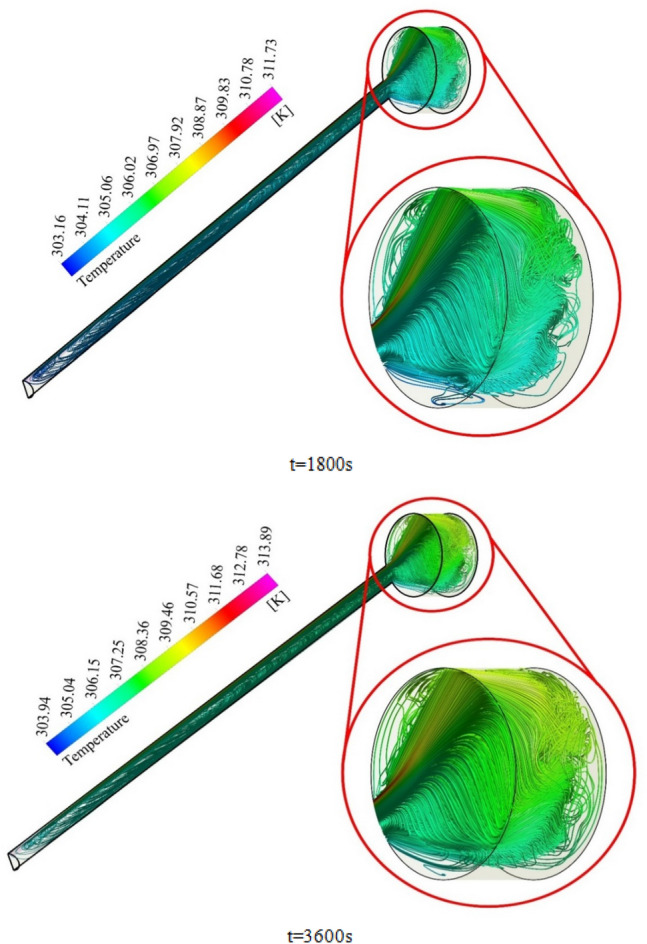
Illustration of temperature for H_2_O.

**Figure 9:**
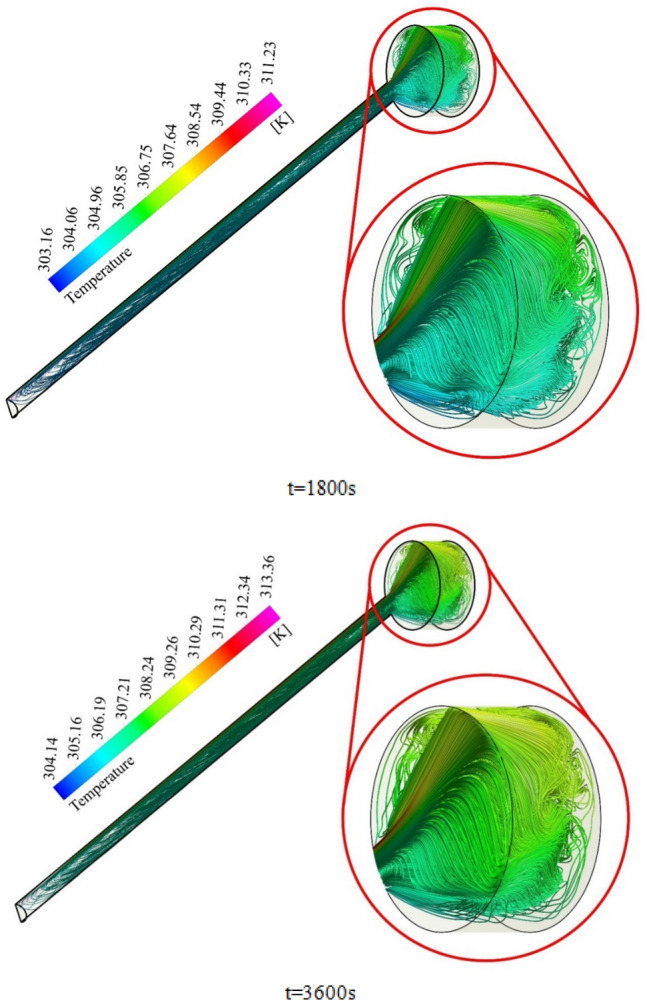
Illustration of temperature for nanofluid.

**Figure 10:**
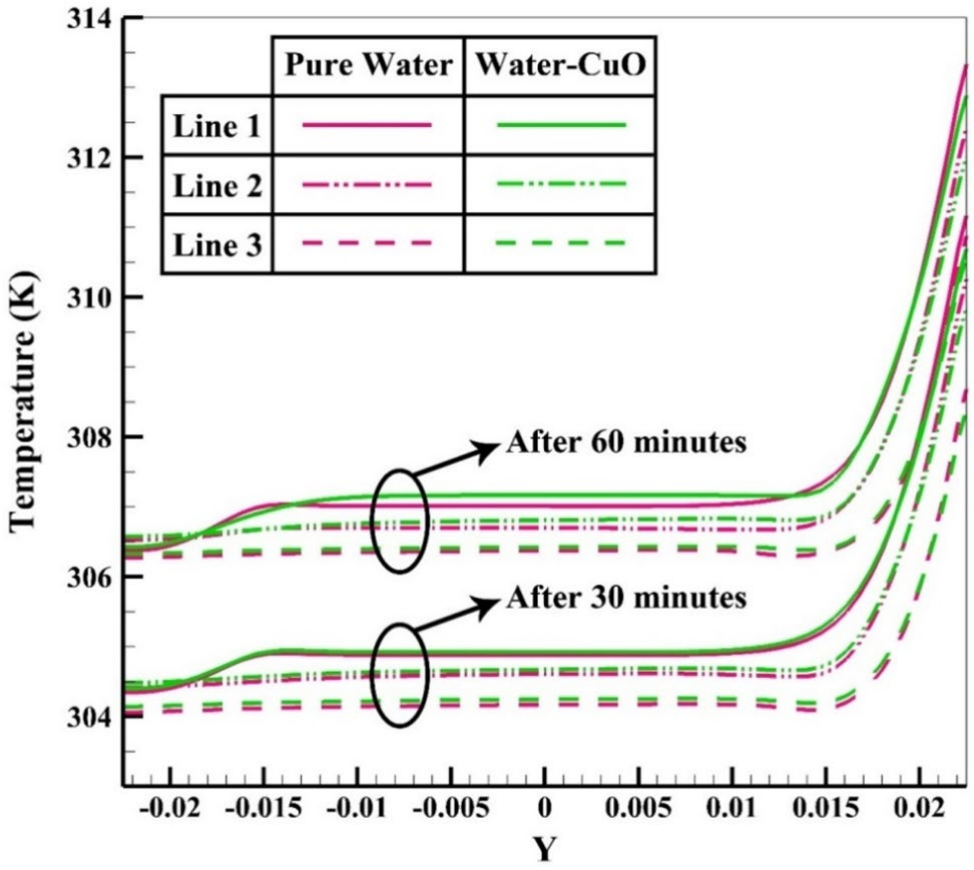
Distribution of temperature at various sections.

**Figure 11:**
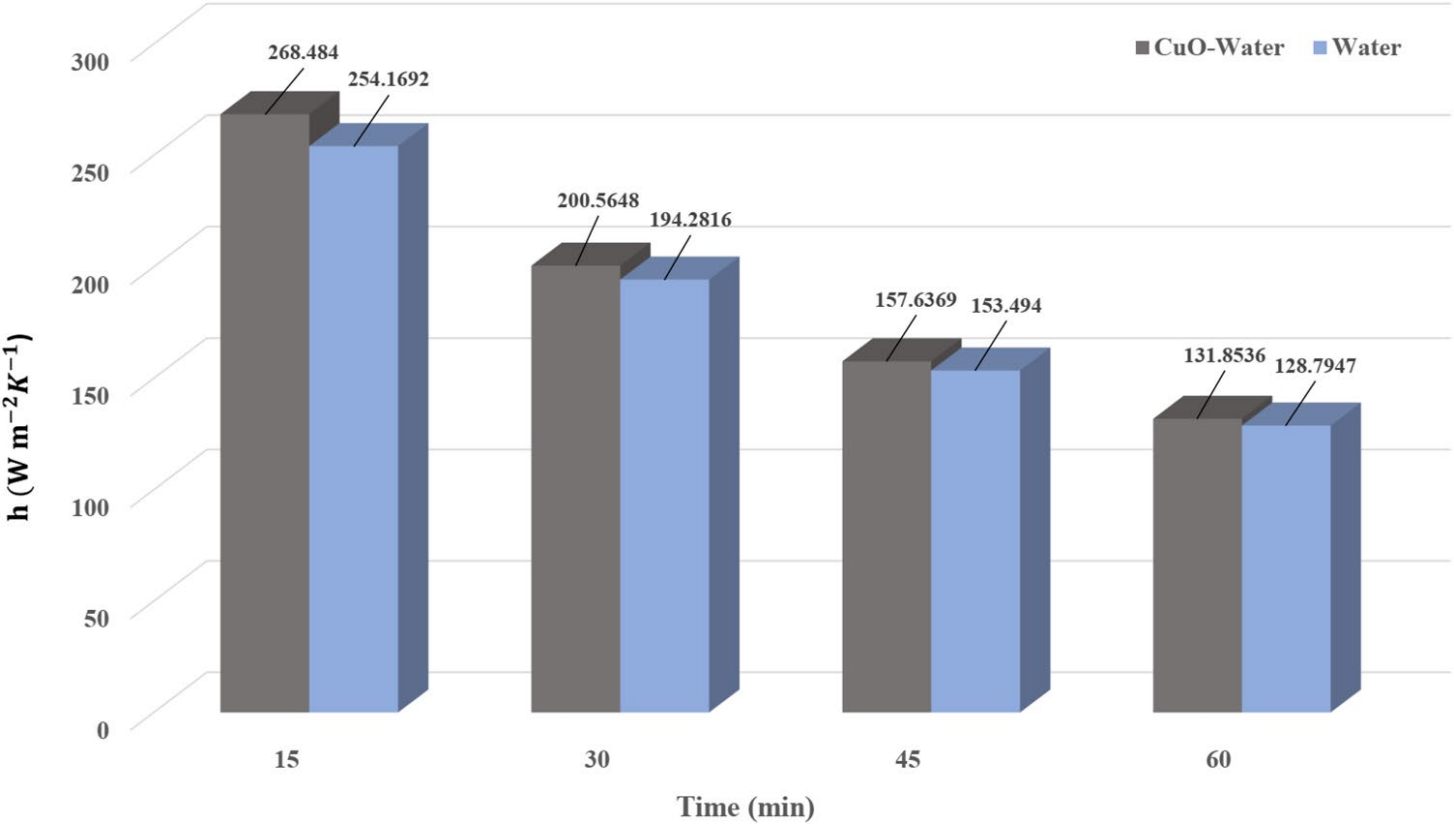
Comparison of performance of unit for nanofluid and water.

To elaborate on the entire natural circulation procedure taking place inside the tube, at the beginning of the process, cold fluid flows from the storage tank toward the absorber’s close-ended region. Meanwhile, the top wall of the absorber, which is at the direct experience to solar radiation, is involved in absorbing and transferring the radiation to the fluid, which migrates inside the collector pipe. After the absorbed heat transmission to the base fluid, the change in density engenders the buoyancy effect, which eventually leads the fluid to experience a free circulation process. Figure 5a,b illustrate the distribution of *V* for water at 0.5 and 1 h after the flow time. To elaborate, the fluid flow inside the tube is divided into layers of fluid with high and low temperatures at the top half and bottom half, which is separated. Figure 6a,b demonstrate the velocity distribution inside the tube and tank for the nanofluid at 0.5 and 1 h after simulation. From a comparative viewpoint, the velocity range for water is marginally lower than the case with using nanofluid. Figure 7a,b comparatively analyze the *V* of different vertical planes of lines 1, 2, and 3 during the passage of flow time. At t = 0.5 h after the flow time, it is observable that a slight discrepancy in velocity values occurs between the two simulated cases in the regions of (15 mm, 20 mm) and (− 0.02 m, − 0.015 m) along the vertical sections for all three lines. The negative velocity values denote the fluid motion toward the pipe’s close end, while positive values refer to the outflow which is moving toward the manifold in the form of natural convection. Among all the cutting sections, the least difference in velocity magnitudes for both cases exists in line 3, which is near the stagnant region on the close-ended side of the absorber, which leads the fluid flow to experience the least velocity value compared to the first line nearby the entry region. On the contrary, the utmost velocity difference of water and nanofluid appertain to line 1, where the fluid flows with the maximum velocity. The last point which can be deduced from Fig. 7a is that line 1 reached the highest peak point of velocity, and as the vertical planes approach the tube’s end, the peak point starts to decrease. This is due to the fact that when water enters the tube sliding layer, which has the lowest speed starts to form in the regions adjacent to the below and up walls of the collector owing to the reverse directions of migration in these regions, which causes in the peak velocity to decline. Figure 7b compares the velocity profiles of the nanofluid and plain water after 1 h. Despite the marginal velocity differences of nanofluid and water in different sections at t = 0.5 h, more differences were observed in the vertical planes of lines 1 and 2, although they remained roughly unchanged in line 3 as the flow time increased up to 1 h. Moreover, the velocity values of water and nanofluid were observed to have more differences between the highest and lowest peak points of velocity compared to other regions in all the sections. What stands out, the outflow velocity of the nanofluid in line 1 (starting at Y = − 0.019) was found to have a higher quantity. All things considered, the velocity values for the nanofluid stood at approximately 40 mm/s in line 1 at the peak point (Y = 0.018) for t = 1 h, which was the highest magnitude among all other sections. The procedure of fluid circulation is shown in temperature distributions for water and nanofluid over the simulation time in Figs. 8 and 9. To be more precise, the bottom region of the tank, which encompasses fluid with lower temperature, flows down to the tube, and the fluid turning back to the manifold flows faster due to the viscosity reduction as a result of temperature enhancement. Furthermore, the free circulation rate faces a reduction nearby the close-ended region of the absorber. Figure 10 depicts the distribution of temperature inside the specified parts of the tube for water and nanofluid after 0.5 and 1 h of flow time. It is deduced that the inflow which is flowing from the manifold toward the tube has a considerably lower temperature than the outflow that returns toward the reservoir. After a 30-min simulation period, the temperature difference between the water and nanofluid remained marginal in all the regions of the cutting planes, while this difference was augmented over the flow time passage. Hence, it can be concluded that using a nanofluid has a pivotal impact on the functionality of the system specifically when the simulation time is extended to 1 day. Among all three sections for the simulated cases, the utmost temperature magnitudes have been obtained for the nanofluid in the first plane at t = 60 min. It is noteworthy to mention that the temperature increment by utilizing nanofluid is observable in the regions from Y = − 0.013 to 0.013 at 1 h after simulation time. The last outstanding feature of this process, as illustrated in the figure, is the intensification of temperature augmentation starting from Y = 0.013 to the top wall of the absorber compared to other regions. This fact can be justifiable when the secondary flow, which originates from the inflow, joins the outflow and its temperature undergoes enhancement. To make a comparison between the two simulated cases and show the merit of employing nanoparticles on the productivity intensification, the "*h*" factor was measured as depicted in Fig. 11. Evidently, the "*h*" was discorved to be higher with the utilization of nanofluid than that of the conventional fluid over the simulation time.

### Entropy generation assessment

**Figure 12:**
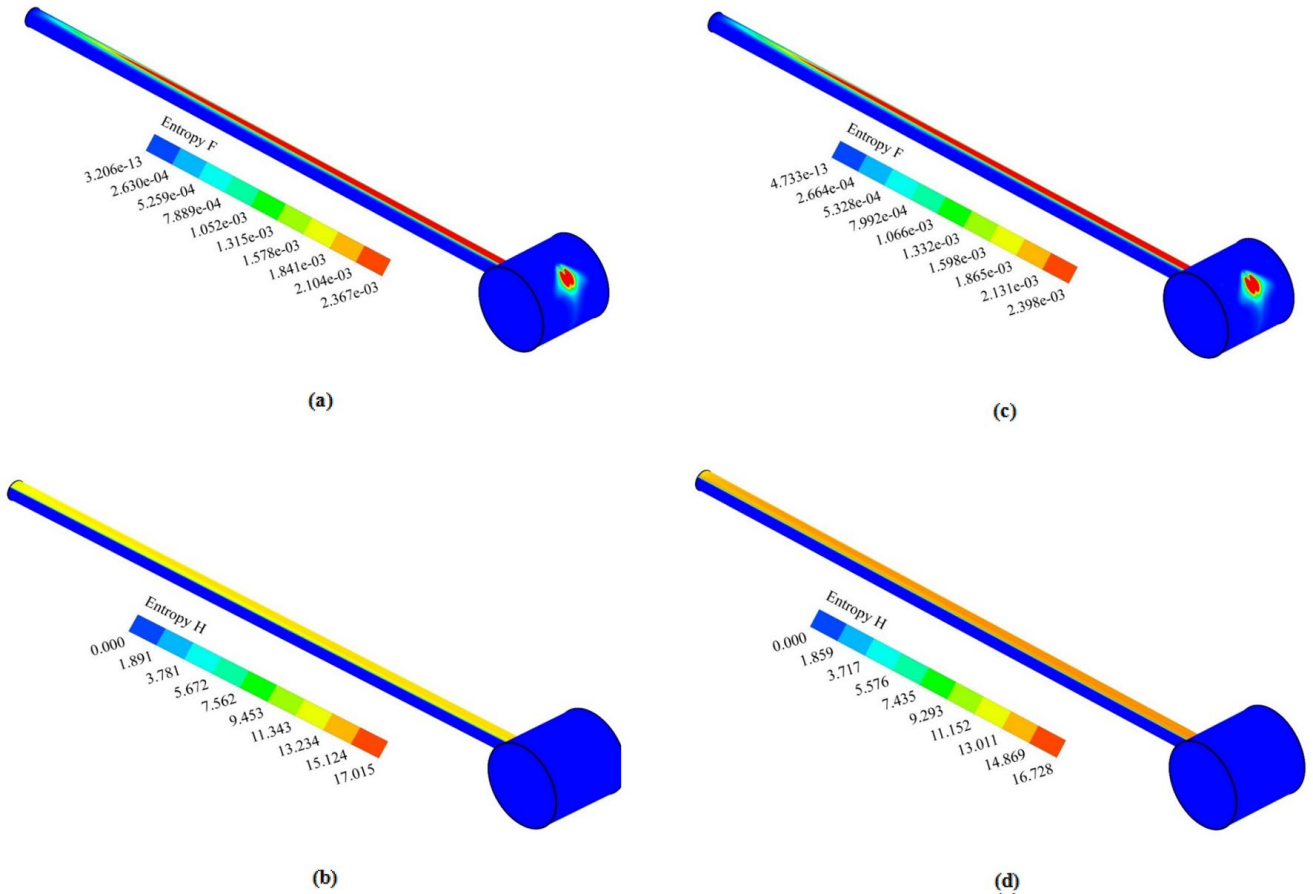
Distribution of S_gen,f_ (**a**) t = 30 and (**c**) t = 60 min, S_gen,th_ (**b**) t = 30 and (**d**) t = 60 min for pure water.

**Figure 13:**
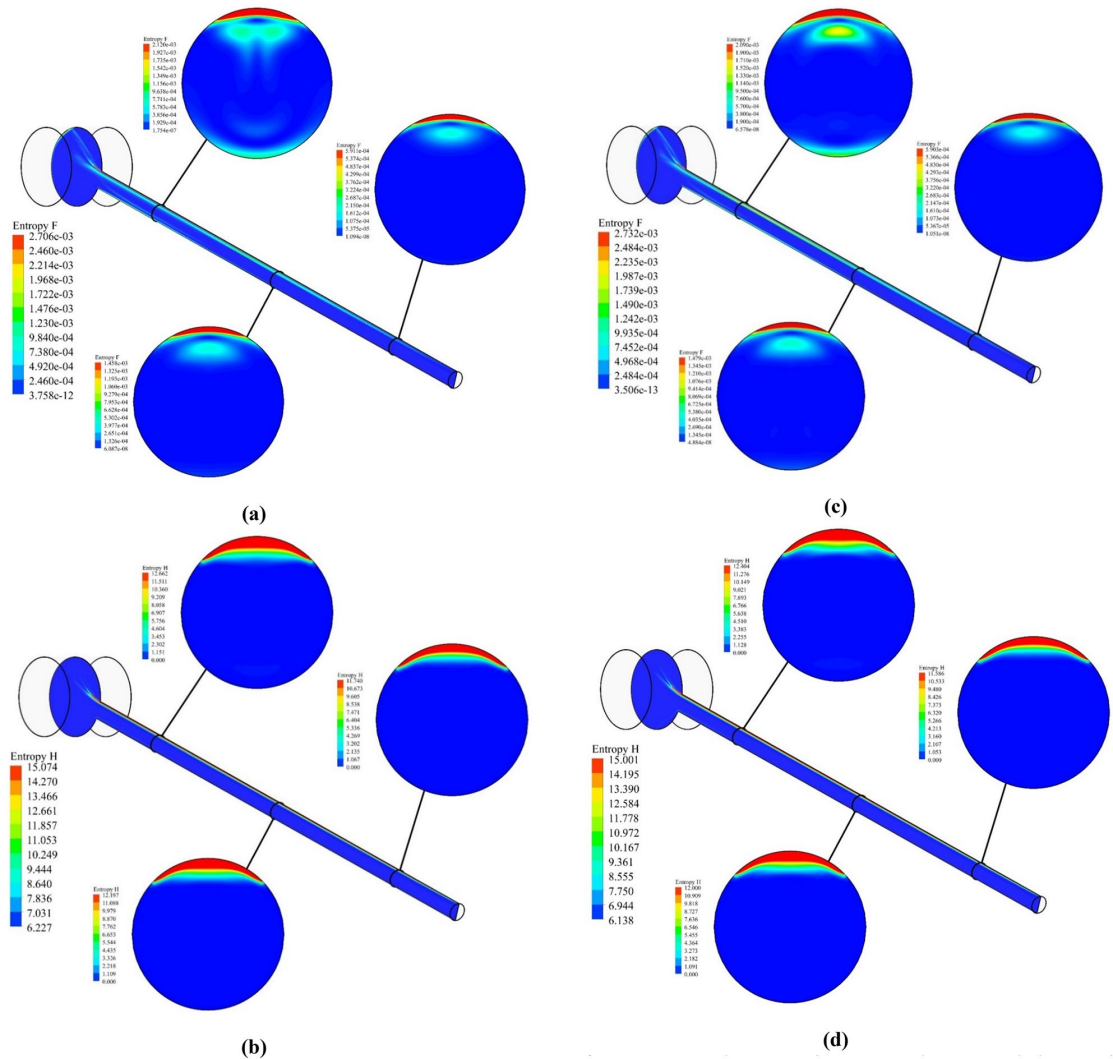
Distribution of S_gen,f_ (**a**) t = 30 and (**c**) t = 60 min, S_gen,th_ (**b**) t = 30 and (**d**) t = 60 min for nanofluid.

**Figure 14:**
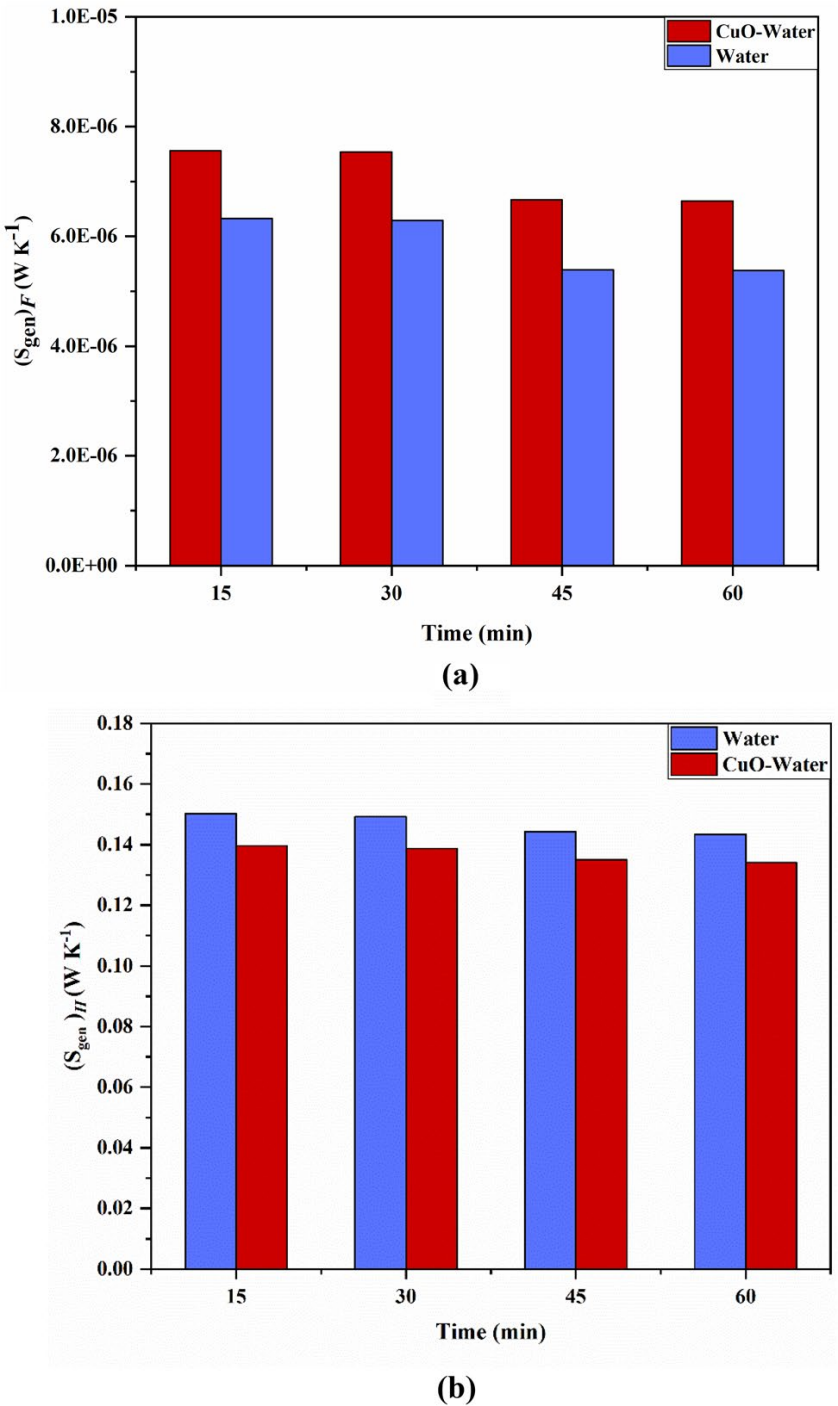
Comparison of (**a**) fluid friction irreversibility and (**b**) heat transfer irreversibility for water and nanofluid.

Besides the thermal assessment aimed at exploring the impact of nanofluid in the collector, an entropy generation analysis is provided for both cases over the simulated flow time to compare the irreversibility in the system under the simulated cases. Fluid friction (${S}_{gen,f}$) and heat transfer (${S}_{gen,th}$) were two factors of entropy generation, which has been considered in this study using the formulation of Eq. (14). Figure 12a,c show the entropy generation distribution for pure water due to fluid viscosity, and Fig. 12b,d demonstrate the entropy generation distribution for pure water due to heat transfer throughout the collector tube and manifold at t = 0.5 h and t = 1 h of flow time. It can also be inferred that the tube’s top wall, which is exposed to the constant solar flux, experiences the utmost heat transfer entropy generation by virtue of the maximum temperature gradient among the base fluid and adjacent walls. Additionally, the maximum fluid friction entropy generation is observable nearby the upper wall of the tube and a part of the manifold, where the outflow possesses the maximum velocity magnitude. Figure 13a,c depict the distribution of fluid friction entropy generation for the nanofluid over the 1-h flow time inside the tube. Additionally, it is deduced that the utmost rate of fluid friction irreversibility in cutting plane 1 occurs in the regions next to the top and bottom surfaces of the absorber, in which the intensity of convective rate and the fluid flow velocity are higher than other regions. Since velocity of the fluid starts to decelerate while flowing toward the close-ended zone of the tube, the fluid viscosity irreversibility at the bottom region of the tube gradually fades out as it is described in Fig. 13a,c. Figure 13b,d depict distribution of thermal-driven entropy generation over the flow time for the case with nanofluid. Figure 14a,b illustrate the comparison of the fluid friction entropy generation and the thermal irreversibility for water and the nanofluid over the simulation time. It is observable that the use of nanofluid leads the fluid friction irreversibility to enhance by 23%, whereas the irreversibility originated from the heat transfer reduced by 6.3% (0.143–0.134 W/K) by utilization of CuO nano-powders with a volume fraction of 5% after 1 h of flow time. Heat transfer irreversibility reduction by utilization of the nanofluid belies in the fact that the ΔT is lower, while the connection improvement occurs in the system with the aid of nanofluid. Turning to the other side, pressure drop increases when the nanofluid is used in the system, which in turn leads to fluid friction augmentation. Notably, the irreversibility owing to heating is considerably bigger than the fluid-viscosity-driven entropy generation when the fluid possesses low velocity, which shows the irrelevancy of the fluid flow viscous stresses in light of the buoyancy-driven nature of the fluid structure within the absorber tube.

## Conclusion

The lack of meticulous studies fixated on the thermodynamic scrutinization of evacuated tube collectors by the presence of nanofluids as an efficacious working fluid for improving the thermal treatment of the system was the main reason this study was carried out. Moreover, the proposed system has undergone thermal evaluation to analyze the fluid structure inside the system and the desirability of the nanofluid flow to enhance the functionality of the collector. The collector tube’s inclination angle tube was fixed at 45 $^\circ$ as the optimum angle, which provides a ggreater temperature range inside the tank. To verify the outputs of present model, numerical and experimental studies were chosen to certify the authenticity of this research and its results. The inclusion of CuO nanomaterial at a 5% volume fraction in plain water was selected in this study according to its merit and had the utmost impact on the collector’s thermal performance improvement compared to all other types of nanoparticles, which led to several changes in productivity of unit. First, the inclusion of nano-sized powders caused the mean temperature of the tank to increase. More importantly, as the simulation time increases from 0 to 3600 s, ΔT among the case with nanofluid and the case with pure H_2_O starts to enhance. Second, the velocity magnitude for the nanofluid was found to be 40 mm/s in line 1 at the peak point in an hour, which was the highest value among all other cutting sections. In addition, to authenticate the productivity improvement of unit by the employ of nanomaterial, a comparison was drawn in terms of "*h*" between the two simulated cases which denote the thermal competency of the provided nanomaterial in proposed system. The convective coefficient was obtained to have higher values over the simulation process with the utilization of CuO by virtue of the effectual thermal characteristics of the prepared fluid when coalesced with the nanoparticles, homogenously. Ultimately, the entropy generation examination as the main objective of this study has shown that adding CuO nanoparticles is a contributing factor, affecting the heat transfer irreversibility to endure a reduction when compared to the conventional fluid. In detail, the irreversibility originated from the heat transfer reduces around 6% with the existence of CuO-${\mathrm{H}}_{2}\mathrm{O}$ nanofluid after the 60-min simulation time. This follows from the fact that finite temperature discrepancy has a lower range when the collector witnesses the thermal performance improvement. On the contrary, the irreversibility originated from the fluid viscosity surges approximately 23% as an outcome of augmentation in the ΔP while suspending nano-sized powders within testing fluid. S_gen_ owing to the fluid viscosity was found to have minuscule values in both simulated cases, which shows the irrelevancy of the fluid flow viscous stresses in light of the buoyancy-driven nature of the fluid flow within the solar unit. All in all, a higher total entropy generation rate was obtained for the conventional type of collector with pure water.

## Abbreviations

AGETC: All glass evacuated tube collector
BA: Boussinesq approximation
FPC: Flat plate collector
VPT: Variable properties temperature
ETSC: Evacuated tube solar collector

## Symbols

*b*: Manifold length (mm)
*D*: Manifold diameter (mm)
*d*: Absorber diameter (mm)
*g*: Gravitational acceleration (m/s^2^)
S_*gen*_: Total entropy generation (W/K)
*T*_*b*_: Bulk temperature (K)
*T*_*w*_: Wall temperature (K)
*T*_*ave*_: The average temperature (K)
*t*: Time (s)

## Greek

*ρ*_0_: Density of fluid at initial temperature (kg/m^3^)
*μ*: Dynamic viscosity (Pa s)

## Subscripts

*l*: Local
*nf*: Nanofluid
*s*: Nanoparticle
*f*: Fluid

## References

[1] Martinopoulos G, Tsilingiridis G, Kyriakis N (2013). Identification of the environmental impact from the use of different materials in domestic solar hot water systems. Appl. Energy.

[2] Comodi G, Bevilacqua M, Caresana F, Pelagalli L, Venella P, Paciarotti C (2014). LCA analysis of renewable domestic hot water systems with unglazed and glazed solar thermal panels. Energy Proced..

[3] Shah TR, Ali HM (2019). Applications of hybrid nanofluids in solar energy, practical limitations and challenges: A critical review. Sol. Energy.

[4] Coimbra J, Almeida M (2013). Challenges and benefits of building sustainable cooperative housing. Build Environ..

[5] Eidan AA, AlSahlani A, Ahmed AQ, Al-fahham M, Jalil JM (2018). Improving the performance of heat pipe-evacuated tube solar collector experimentally by using Al_2_O_3_ and CuO/acetone nanofluids. Sol. Energy.

[6] Chopra K, Pathak AK, Tyagi VV, Pandey AK, Anand S, Sari A (2020). Thermal performance of phase change material integrated heat pipe evacuated tube solar collector system: An experimental assessment. Energy Convers. Manage..

[7] Jowzi M, Veysi F, Sadeghi G (2019). Experimental and numerical investigations on the thermal performance of a modified evacuated tube solar collector: Effect of the bypass tube. Sol. Energy.

[8] Yurddaş A (2020). Optimization and thermal performance of evacuated tube solar collector with various nanofluids. Int. J. Heat Mass Transf..

[9] Zhang X, You S, Xu W, Wang M, He T, Zheng X (2014). Experimental investigation of the higher coefficient of thermal performance for water-in-glass evacuated tube solar water heaters in China. Energy Convers. Manage..

[10] Naik BK, Bhowmik M, Muthukumar P (2019). Experimental investigation and numerical modelling on the performance assessments of evacuated U-tube solar collector systems. Renew. Energy.

[11] Alfaro-Ayala JA, López-Núñez OA, Gómez-Castro FI, Ramírez-Minguela JJ, Uribe-Ramírez AR, Belman-Flores JM (2018). Optimization of a solar collector with evacuated tubes using the simulated annealing and computational fluid dynamics. Energy Convers. Manage..

[12] Yao K, Li T, Tao H, Wei J, Feng K (2015). Performance evaluation of all-glass evacuated tube solar water heater with twist tape inserts using CFD. Energy Proced..

[13] Essa MA, Mostafa NH (2017). Theoretical and experimental study for temperature distribution and flow profile in all water evacuated tube solar collector considering solar radiation boundary condition. Sol. Energy.

[14] Alfaro-ayala JA, Martínez-rodríguez G, Picón-núñez M, Uribe-ramírez AR, Gallegos-muñoz A (2015). Numerical study of a low temperature water-in-glass evacuated tube solar collector. Energy Convers. Manage..

[15] Mazarrón FR, Porras-Prieto CJ, García JL, Benavente RM (2016). Feasibility of active solar water heating systems with evacuated tube collector at different operational water temperatures. Energy Convers. Manage..

[16] Sharafeldin MA (2018). Evacuated tube solar collector performance using CeO_2_/water nano fluid. J. Clean. Prod..

[17] Ozsoy A, Corumlu V (2018). Thermal performance of a thermosyphon heat pipe evacuated tube solar collector using silver-water nanofluid for commercial applications. Renew. Energy.

[18] Gan YY, Ong HC, Ling TC, Zulkifli NWM, Wang CT, Yang YC (2018). Thermal conductivity optimization and entropy generation analysis of titanium dioxide nanofluid in evacuated tube solar collector. Appl. Therm. Eng..

[19] Dehaj MS, Mohiabadi MZ (2019). Experimental investigation of heat pipe solar collector using MgO nanofluids. Sol. Energy Mater. Sol. Cells.

[20] Yan S, Wang F, Shi ZG, Tian R (2017). Heat transfer property of SiO2/water nanofluid flow inside solar collector vacuum tubes. Appl. Therm. Eng..

[21] Sharafeldin MA, Gróf G, Abu-Nada E, Mahian O (2019). Evacuated tube solar collector performance using copper nanofluid: Energy and environmental analysis. Appl. Therm. Eng..

[22] Bejan A (1979). A study of entropy generation in fundamental convective heat transfer. J. Heat Transfer..

[23] Kaushik SC, Reddy VS, Tyagi SK (2011). Energy and exergy analyses of thermal power plants: A review. Renew. Sustain. Energy Rev..

[24] López A, Ibáñez G, Pantoja J, Moreira J, Lastres O (2017). Entropy generation analysis of MHD nanofluid flow in a porous vertical microchannel with nonlinear thermal radiation, slip flow and convective-radiative boundary conditions. Int. J. Heat Mass Transf..

[25] Leong KY, Saidur R, Mahlia TMI, Yau YH (2012). Entropy generation analysis of nanofluid flow in a circular tube subjected to constant wall temperature. Int. Commun. Heat Mass Transf..

[26] Ramírez-Minguela JJ, Alfaro-Ayala JA, Rangel-Hernández VH, Uribe-Ramírez AR, Mendoza-Miranda JM, Pérez-García V (2018). Comparison of the thermo-hydraulic performance and the entropy generation rate for two types of low temperature solar collectors using CFD. Sol. Energy.

[27] Xu L, Wang Z, Yuan G, Li X, Ruan Y (2012). A new dynamic test method for thermal performance of all-glass evacuated solar air collectors. Sol. Energy.

[28] Sato AI, Scalon VL, Padilha A (2012). Numerical analysis of a modified evacuated tubes solar collector. Renew. Energy Power Qual. J..

[29] Advanced Engineering Thermodynamics-Adrian Bejan-Google Books n.d. https://books.google.com/books?hl=en&lr=&id=j0zSDAAAQBAJ&oi=fnd&pg=PR17&dq=Bejan+A.+Advanced+engineering+thermodynamics:+John+Wiley+%26+Sons,+2016.&ots=2wVXZeCtQp&sig=iMwjebtnWK5Q9IYA6Au6iDOEyPA#v=onepage&q&f=false. Accessed 11 Nov 2021.

[30] Bellos E, Tzivanidis C, Papadopoulos A (2019). Enhancing the performance of a linear Fresnel reflector using nanofluids and internal finned absorber. J. Therm. Anal. Calorim..

[31] Kim D, Kwon Y, Cho Y, Li C, Cheong S, Hwang Y (2009). Convective heat transfer characteristics of nanofluids under laminar and turbulent flow conditions. Curr. Appl. Phys..

[32] Bejan A, Kestin J (1983). Entropy generation through heat and fluid flow. J. Appl. Mech..

[33] Zimparov V (2001). Extended performance evaluation criteria for enhanced heat transfer surfaces: Heat transfer through ducts with constant heat flux. Int. J. Heat Mass Transf..

[34] Mercan M, Yurddaş A (2019). Numerical analysis of evacuated tube solar collectors using nanofluids. Sol. Energy.

